# Theorizing the complexity of HIV disclosure in vulnerable populations: a grounded theory study

**DOI:** 10.1186/s12889-018-5073-x

**Published:** 2018-01-19

**Authors:** Subash Thapa, Karin Hannes, Anne Buve, Shivani Bhattarai, Catharina Mathei

**Affiliations:** 1 Department of Public Health and Primary care, KU Leuven, Kapucijnenvoer 33, 3000 Leuven, Belgium; 2 Department of Public Health, Institute of Tropical Medicine, Nationalestraat 155, 2000 Antwerp, Belgium; 3 Centre for Sociological Research, Faculty of Social Sciences, KU Leuven, Parkstraat 45, 3000 Leuven, Belgium; 4 Department of Public Health, Nobel College Pokhara University, Kathmandu, 44601 Nepal

**Keywords:** Community self-coping of HIV, Forced disclosure, Grounded theory, HIV disclosure, HIV stigma, Public health system

## Abstract

**Background:**

HIV disclosure is an important step in delivering the right care to people. However, many people with an HIV positive status choose not to disclose. This considerably complicates the delivery of adequate health care.

**Methods:**

We conducted a grounded theory study to develop a theoretical model explaining how local contexts impact on HIV disclosure and what the mechanisms are that determine whether people choose to disclose or not. We conducted in-depth interviews among 23 people living with HIV, 8 health workers and 5 family and community members, and 1 community development worker in Achham, Nepal. Data were analysed using constant-comparative method, performing three levels of open, axial, and selective coding.

**Results:**

Our theoretical model illustrates how two dominant systems to control HIV, namely a community self-coping and a public health system, independently or jointly, shape contexts, mechanisms and outcomes for HIV disclosure.

**Conclusion:**

This theoretical model can be used in understanding processes of HIV disclosure in a community where HIV is concentrated in vulnerable populations and is highly stigmatized, and in determining how public health approaches would lead to reduced stigma levels and increased HIV disclosure rates.

## Background

HIV disclosure is a process to inform others about one’s HIV positive status by the person himself or by a third party with or without consent [1]. The World Health Organization (WHO) and the Joint United Nations Program on HIV/AIDS (UNAIDS) particularly promote HIV disclosure [1]. After the disclosure, people living with HIV (PLWH) are more likely to access treatment and live a healthier life, experience a normal romantic life as much as possible taking into account prevention strategies and positively contribute to the community [2, 3]. However, it is crucially important to reduce social stigma against PLWH in order to prevent HIV transmission [1, 2].

Stigma is a social process, experienced or anticipated, characterized by exclusion, rejection, blame, or devaluation that results from experience, perception, or reasonable anticipation of an adverse social judgement about a person or group [4]. UNAIDS defined HIV stigma as a process of devaluation of people either living with or associated with HIV infection [5]. PLWH have been stigmatized because the disease is generally perceived as dangerous, contagious, and associated with behaviours outside of social norms [4]. HIV stigma may have serious consequences, such as loss of friendship and family ties, dismissal from school and occupation, and denial from health care [6, 7]. HIV stigma has been shown to affect multiple HIV-related health behaviours and outcomes (e.g., accessing treatment and testing services) in people living or associated with HIV and the general population [8]. Because of HIV stigma, one-third of individuals testing positive with HIV globally do not disclose their HIV status [6].

Obermeyer (2011) has stated that a majority of PLWH would eventually disclose their HIV status at one point of their life [2]. Early in the epidemic, it was predicted that people diagnosed with HIV were disclosed as their disease progressed and they became ill and symptomatic [9]. Today, because of the availability of highly active antiretroviral therapy, individuals live longer, often asymptomatic lives and an HIV infection has become a long term manageable condition [10]. Even so, to access the treatment, one has to test for HIV and needs to disclose the positive test result, at least to a health worker. HIV disclosure may not happen as one-time full disclosure to everyone, and the decisions about when, to whom, and how to disclose may gradually increase over time [5]. These mechanisms are important to understand in the context of designing and delivering optimal health care to PLWH and should therefore be unpacked.

We developed a theory to increase our understanding on the contextual factors and mechanisms that influence HIV disclosure. This theory is presented as an organized and systematic set of interrelated concepts that helps to develop laws of general understanding by determining whether, why and how disclosure takes place and to address what types of support people, families and communities need [11]. The model is believed to guide future research projects on HIV disclosure, and to facilitate the development and implementation of robust interventions to reduce stigma and potentially increase the rates of HIV disclosure [12].

## Methods

We opted for a grounded theory design to study HIV disclosure processes in vulnerable populations in Nepal. Grounded theory is generally referred to as an inductive research process that leads to the systematic development of a theoretical model to explain behavioral patterns and processes in social settings linked to the phenomenon under study [13]. In our case, the phenomenon central to our study is the process of HIV disclosure. In grounded theory, rather than introducing a set of preconceived theoretical ideas or hypotheses to guide data-collection and analysis, the building blocks for the emerging theory are generated through a process of simultaneous data collection and analysis. Data collection and analysis continues until a saturation point is reached and the developed theory is no longer challenged by new, potentially conflicting data collected [14].

### Study setting

This study was conducted in Achham, one of the districts with the highest prevalence of HIV in Nepal [15, 16]. An important contextual factor that contributes to the high prevalence of HIV infection in Achham is men’s seasonal migration to India [17]. In Nepal, the HIV epidemic is concentrated among vulnerable sub-groups in the population, such as male labor migrants, female sex workers, and men having sex with men, with labour migrants as the sub-population most affected by HIV [15]. The prevalence of HIV infection among the Nepalese labour migrants is 2.8% and among their wives is 0.8% while the prevalence among the general male and female populations is less than 0.5% [18, 19].

Several studies have shown that persons in some concentrated epidemic settings have expressed more stigma against PLWH than persons in settings with generalized HIV epidemics [20, 21]. In concentrated epidemic settings, there is lesser exposure to HIV-related health education and stigma reduction programs among general population, which may have an influence on increased negative attitudes towards PLWH [20]. In a community where heterosexual sex is the predominant route of infection, HIV stigma is mainly attributed to infidelity and sex work [22]. And thus, the general population might fear transmission from the affected sub-populations and therefore, might have a different opinion about these population groups that are disproportionately affected by HIV. Therefore, we conducted this study in Achham, Nepal to understand whether, why and how HIV disclosure takes place in a setting where HIV is concentrated among sub-populations. The findings of this study may help program managers and policymakers to develop effective strategies to reduce stigma and to prevent new infections among the vulnerable populations and thus, to lower the total burden of HIV infection in Nepal.

### Sampling

Theoretical sampling was employed to identify and select the participants. “Theoretical sampling is the process of data collection in which the researcher simultaneously collects, codes, and analyses data and decides what data to collect next and where to find them” [14]. The preliminary data analysis helped us to decide on the type of profile that could best increase our understanding of the theoretical leads derived from the field and helps to inform our theory development phase. Therefore, the grounded theory study entailed studying: health workers, PLWH, their family, community members without the disease, and a community development worker. The inclusion criteria were being at least 18 years old and showing willingness to participate in the study after being informed about the study purpose.

#### Health workers

We approached three different types of health workers, namely health workers without the disease, health workers living with HIV and female community health volunteers without the disease. The reason behind inviting health workers without the disease for an interview was that they may have witnessed people’s experiences of HIV disclosure and may be better placed to neutrally describe what actually happens in the move from non-disclosure to disclosure. The health workers living with HIV may share their own experiences of living with HIV and dealing with other people without the disease in their personal and professional life.

The female community health volunteers were local women trained to provide some basic health services (e.g. providing health education or distributing condoms) in the community and to refer the community people to the nearby health centre [15]. We assumed that these women would be aware of the experiences of both PLWH and the opinions and experiences of family and community members in relation to HIV disclosure and stigma*.*

#### People living with HIV

We considered PLWH who had disclosed publicly, who had disclosed only to their family or partner and who had disclosed only to the health worker, for our sample expecting that these different groups would experience things differently. The people having different disclosure statuses described their experiences of disclosure and experiences of benefits and harms in-depth.

All the PLWH were recruited in the Mangalsen Hospital, the only governmental hospital providing treatment and care to PLWH in Achham. The Anti-Retroviral Therapy (ART) counsellor working in the Mangalsen Hospital asked some selected PLWH who came to the hospital to receive the treatment whether they had disclosed or kept secret about their HIV status to their spouse, family members and the community members and whether they would be interested to talk to a researcher.

#### Family and community members without HIV

In order to fully understand disclosure behaviour and stigma as the central concepts that emerged from the data, we had to speak to people without HIV in the direct environment of the PLWH to investigate how their behavioural patterns and ideas might influence the other’s behaviour. Therefore, we consulted the health workers who participated in the interview to identify and recruit the family and community members of PLWH in the study. The family members described the disclosure experiences of PLWH in the family, their relationship before and after the disclosure, discrimination experiences and the role they have played to support the PLWH. The community members provided information about their general opinion toward HIV and PLWH, how they would know about one’s HIV status, their experiences with the person and how other people think and behave in the community.

We also asked one community development worker without the disease to participate in the study. Community development workers are appointed by the government to work for planning, implementing and evaluating the local development works in the community. The community development worker gave information about the help and support provided to PLWH and described his experiences of working with them.

Altogether, there were 37 participants recruited in the study that included 23 PLWH, 8 health workers and 5 family and community members, and 1 community development worker (see Table 1). The majority of the participants were male (54%), were literate (65%), belonged to higher caste (62%), and were living with HIV (68%). The mean age of the participants was 33 years.

**Table 1:** Socio-demographic characteristics of the participants

Participants	*n*	< 33 years	> 33 years	Male	Female	Literate	Illiterate	Higher Caste^a^	Lower Caste^a^
		*n* (%)	*n* (%)	*n* (%)	*n* (%)	*n* (%)	*n* (%)	*n* (%)	*n* (%)
People living with HIV	23	8 (34.8)	15 (65.2)	10 (43.5)	13 (56.5)	11 (47.8)	12 (52.2)	11 (47.8)	12 (52.2)
Health workers	8	7 (87.5)	1 (12.5)	5 (62.5)	3 (37.5)	8 (100.0)	0 (0.0)	8 (100.0)	0 (0.0)
Family/community members	6	4 (66.7)	2 (33.3)	5 (83.3)	1 (16.7)	5 (83.3)	1 (16.7)	4 (66.7)	2 (33.3)

^a^Caste is a socially constructed stratum and is ascribed by birth. It affects food practices, occupations, culture, marriage and family life. People who belonged to lower caste are generally considered less privileged

### Data collection

At first, we set the agenda in terms of the topics to be covered and developed the questions based on the following topics: perception about HIV, experiences on living with (a person with) HIV, disclosure behaviour and benefits and harms experienced or perceived. The question guide was prepared first in English and then translated into Nepali. The question guide used open-ended topical prompts and probes to gain the desired depth of information and was developed through group discussion among the team members. The question guide was then again reviewed by the health workers in the Mangalsen hospital to check whether the words and phrases were appropriate to the local context. Considering the fact that the women of Achham would not normally talk openly with an unfamiliar man, a female researcher trained in qualitative research was hired to conduct the in-depth interviews with female participants.

Initially, we interviewed a health worker in the Mangalsen hospital and then continued our interviews with other PLWH of different disclosure status. We assumed that face-to-face interviewing on an individual basis would be comfortable for the participants to discuss about their experiences. However, after conducting some interviews, we noticed that some of the participants living with HIV did not feel comfortable to talk about their stories in a face-to-face interview because of the sensitivity of the topic. Then, we invited two or three PLWH to participate in a group interview. Two group interviews were conducted with women living with HIV, one with men living with HIV and three mixing both women and men. We assumed that the variations in the composition of the group would lead to variations in discussion and responses.

Additional participants were interviewed based on the preliminary data analysis. The participant’s responses determined whether to consider new topics to introduce or adjust the existing question guide. For example, after we learned about gossiping and rumours, we interviewed the community members who shared information about potential negative outcomes of disclosure in the community. After we learned about cultural stigma, we interviewed the religious leader and some community members to further describe it in-depth.

Altogether, we conducted 23 individual interviews and 6 group interviews from November 2015 to February 2016. All the interviews with PLWH and the family members took place in one of the rooms of the Mangalsen hospital. Some interviews with family and community members were also conducted at community health centers and at their houses. A written informed consent procedure was completed with each participant. All the interviews were audiotaped with permission and transcribed verbatim. On average, both the individual interviews and group interviews lasted about 1 h.

### Positional stance

The first author was a Nepalese man familiar with the local dialect and culture. He had experiences of conducting other research projects with PLWH in Nepal. The female researcher was a Nepalese woman trained as a research assistant and holding a bachelor’s degree in public health. She was born and socialized near to Achham and was familiar with the local dialect and culture. Both researchers were born and raised in a community where HIV is highly stigmatized, which helped them to understand the sensitivity involved in discussing about HIV status. Both wrote down memos during and after each interview and discussed about their interpretations with the health workers from the Mangalsen hospital.

### Analysis

Data for analysis included transcripts of audio recordings from the in-depth interviews. The female researcher transcribed and translated all the recordings from Nepalese language to English, and the first author checked for the quality. Necessary reconciliations were made after the discussion. Data analysis were carried out using constant comparative method, performing three levels of open, axial, and selective coding [14].

At first, open coding was performed to generate initial codes from the transcripts. The transcripts were first read thoroughly and then each line was labelled with codes expressing a concept related to the research topic, which produced nearly 90 open codes (see Table 2). The codes were verified if they reflected informants’ ideas, were examined for overlap and then collapsed into 24 broader codes. These codes were then organized into 7 overarching categories. Coded texts were extracted and organized by category and explanations were sought for the similarities and differences between each other.

**Table 2:** Coding tree

Categories	Broader codes	Codes
Social stigma	Socio-cultural beliefs	Sexual taboo; Cultural misbeliefs
	Misconception	Wrong ideas about mode of HIV transmission; Wrong perceptions about people living with HIV (PLHA); Criminalization of HIV; Perceived similarity between HIV and leprosy
	Social curiosity	Social attributes of HIV; Strategies for suspicion
Discrimination	Exclusion	Exclusion in the family; Peer group exclusion; Social exclusion, Children drop out from school,
	Verbal/ physicial assualt	Said bad words; Teasing; Being blamed for transmitting HIV; Women beaten by their husband’s
	Denial from love and care	Relationship break up; Left to die without care; Denial from health care
	Cultural discrimination	Regarded impure; Cultural exclusion; No cremation rituals followed
Negative emotions	Fear	Fear of discrimination; Fear of forced disclosure; Fear of discrimination to family members; Fear of losing respect
	Shame	Ashamed of being HIV infected; Regarding oneself as a bad person
	Mistrust	Lack of trust to family members; Lack of trust to health workers
Public health initiatives	Health care access	HIV-related health services; General health services for PLHA
	Incentives	Incentives for testing for general population; Incentives for PLHA; Social support for PLHA
	Involvement	Community developmental; Community organizations; HIV-related committees; Political groups; Health service delivery; Income generation
Mediating factors	Relationship with health workers	Trust; Adherence; No discrimination by health workers; Health workers helping with disclosure
	Knowledge	Availability of treatment; Mode of transmission; Benefits of disclosure; Negative consequences of non-disclosure
	Perceived income	Jobs and better positions; Access to loans; Incentives
	Perceived social support	Care during illnesses; Perceived solidarity; Children taken care by others after the death; Help and support
Empowerment	Political empowerment	Representation in political parties; Inclusion in developmental works; Representation in school management committee; Formation of organizations and committees
	Economic Empowerment	Jobs; Access to loans; Incentives; Involved in private business and farms
	Social empowerment	Social networking; Identity; Community organizing; Fighting against discrimination; Normalization of HIV; Peer support
	Personal empowerment	Reduced mental stress; Self care; Self determination; Introducing oneself as an HIV infected person; Activism
Mechanism of disclosure	Disclosure status	Self disclosure; Disclosure with consent; Forced disclosure
	Disclosure status	Undisclosed; Disclosed to health workers; Disclosed to family members; Disclosed to general public
	Disclosure avoidance behavior	Lying; Hinting; Avoiding contacts with people living with HIV; Do not seek health care; Seeking traditional healers; People secretly taking medicines; Late testing

In axial coding, connections within and between categories were explored to develop a more abstract level of conceptualization. For instance, concepts, such as ‘public health initiatives as a facilitator of disclosure’ and ‘stigma as a barrier’ were explored. Memos were written to document the conceptual and theoretical ideas that emerged when exploring the connections within and between categories. Visual models were developed to facilitate the visualization of data for better conceptualization. The connections within and between categories were evaluated and discussed within the research team that also helped to determine missing concepts and to identify additional participants. The leads from the initial analysis informed the process of simultaneous sampling, data collection and analysis, and this process was repeated until data saturation was reached.

Next, in the selective coding phase we extracted two core storylines centred around the idea of HIV disclosing systems: ‘community self coping with HIV’ and ‘public health system to control HIV’. This allowed us to build a theoretical model. Subsequent changes based on the discussions and agreements among the team members were made to come up with the final theoretical model.

### Controlling for the quality of the study

To minimize the potential negative impact of the lead researcher on the data collection process and the interpretation of the findings we invited an additional female co-worker to assist with these processes. Discussions between both researchers were considered important in deciding what data to collect based on the leads from the preliminary analysis and whom to interview [13]. Within the context of the current study, the first author and the female researcher involved in face-to-face contact with study participants considered the probable ways in which interactions with participants might be influenced by our professional background, experiences and prior assumptions.

In order to ensure the credibility of the findings, member checking was performed to correct for potential under- or over interpretation of collected data in developing the conceptual layers of the study and in building the theoretical model [23]. We further asked three health workers working in the Mangalsen Hospital: the ART counsellor, the HIV program manager and the Public Health officer, to provide input into the development of study materials and the recruitment of the participants. This process insured credibility and consistency of the research findings to the people we interviewed and to the research context.

Peer debriefing was performed with team members with a Western background, who oversaw the data and theory from an outsider’s perspective and provided their reflections and interpretations, based on their readings of the categories and themes evolving from the data [24]. The interpretations provided by the team members who belonged to a completely different socio-cultural context were also helpful to correct for potential over- or under-interpretation and to frame the findings conceptually. Besides, during coding phase, several researchers from different background who were not directly involved in this study were invited to identify and discuss potential codes and categories from the data and to develop the theoretical model. This process might have strengthened credibility and transferability by illuminating the research from a variety of the perspectives from the researchers [24].

Memos were written throughout the data collection and analysis period to document the interview setting, participant’s emotions, direct quotations, the researcher’s reactions, theoretical ideas evolving from exploring the data, the relationship between the codes and categories and the description how they helped to develop the theory grounded in the empirical data [14]. The careful use of memos during analysis, which moved from raw data, through interpretation, to the development of theory, provided a visible ‘audit trail’. An example of the excerpt of the memo is illustrated in Table 3.

**Table 3:** An example of the excerpt of memo

**ᅟ**	**ᅟ**
**Memo 3. Access to health service and disclosure facilitated by the health worker, December 9, 2015** (…) Some people, especially men, are tested positive when they are brought to the hospital by the family members due to some illnesses (*The fever kept on increasing, we had to take him to the hospital)*. In case of a positive test result, the doctor will also suggest the spouse and children to have an HIV test (T*he doctor also asked for my blood for the check up).* In this situation, HIV infected people are helped by the health worker to disclose the HIV positive status to the spouse and family members with an oral consent.	

## Results

**Fig. 1:**
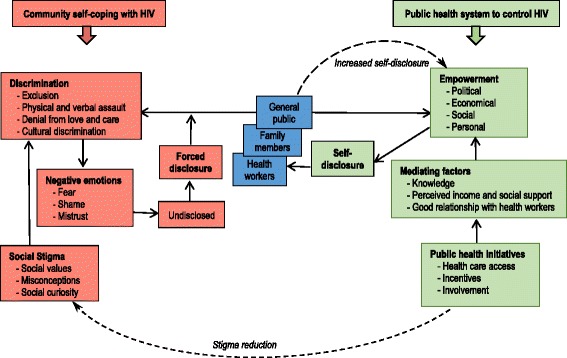
Theoretical model on complexity of HIV disclosure in at-risk populations

The following categories were developed from analyzing the interview transcripts: ‘social stigma’, ‘discrimination’, ‘negative emotions’, ‘public health initiatives’, ‘mediating factors’, ‘empowerment’, and ‘mechanism of disclosure’. Based on the similarity in meaning, we organized and structured the categories into a theoretical model (see Fig. 1), outlining how two dominant systems, namely ‘Community self-coping with HIV’ (red colored boxes) and ‘Public health system to control HIV’ (green colored boxes), independently or jointly, shape factors, mechanisms and outcomes of HIV disclosure.

### Community self-coping with HIV

A community self-coping with HIV is defined as the actions taken by the community to cope with HIV and the perceived threat. These actions include identifying, labelling, devaluing and distancing PLWH in order to reduce the risk of transmission. These coping actions and perceived threats are produced in a community where HIV is highly stigmatised and discriminated.

Community self-coping actions often start with the process of inquiring information about one’s HIV positive status, which is known as social curiosity. The curiosity about HIV among community members is due to misconceptions and perceived threat of HIV. Confirmation of one’s HIV information without his/her consent leads to forced disclosure, which further leads to labelling, neglect and exclusion.

PLWH who have suffered forced disclosure and discrimination, often feel a shame related to them being infected. They strongly feel that they are part of the stigmatized group, which isolates them from the community. The community members who have closely witnessed forced disclosure and discrimination are likely to perceive HIV as a threat for exclusion and engage in actions such as, avoiding PLWH to reduce the risk of HIV transmission. In what follows, we outline components that influence non-disclosure.

#### Social stigma

The majority of participants described HIV as being linked to impurity and extramarital sex, which was considered as a sign of bad behavior in the community. This belief system crucially impacted on some of the rituals in place in the community. Other misconceptions reported by several participants were:

*“HIV is caused due to sin of forefather or curse of God”;*

*“HIV transmits via touching, and sharing food and water and kitchen utensils with an infected person”;*

*“HIV has no cure”;*

*“A man who transmits HIV to his wife is a criminal”;*

*“HIV is as bad as leprosy; that’s why the infected should be kept away from the community”.*

Social curiosity is the act of trying to gain information about one’s HIV positive status. The community members were frequently reported being engaged in observing people who were suspected to be infected with HIV or asking questions to health workers about a person’s HIV positive status. It was generally perceived that men, who had previously worked in India and returned back home due to illness similar to tuberculosis were considered suspicious, particularly when they had frequently been visiting the hospital. Also, community members who were found seeking HIV-related services or staying in the HIV care home or meeting other PLWH or being visited by a health worker from HIV-related organizations were suspected of having an HIV positive status. If a person was known of dying from AIDS, his/her spouse and children were also suspected of having an HIV positive status.

#### Discrimination

Several participants living with HIV reported being discriminated by the family and community members. The most frequently reported forms of discriminations are social exclusion, neglect, verbal assault, physical violence and cultural discrimination.

##### Exclusion

Some of the participants reported that they were forced to live separately, could not share food and drink, and were not allowed to use the the common toilet, and a few also reported being excluded from the family. Some participants living with HIV mentioned being neglected during encounters with other people, and not being visited or not invited to cultural ceremonies. They also mentioned that their children were expelled from school. One widowed woman (aged 22-years old) mentioned that, after the community members found out about her HIV status, she along with her children was forced to leave the community. She put it this way:

*“After my husband died from AIDS, the community people threatened me to leave the community. They said that I could not stay there any longer. I came to my father’s place with all my belongings. He built a small hut for me and my children.”*

*“In the past days, in our village, people used to isolate the people with leprosy (taken away from the society and was forbidden to re-enter the society). They also did the same for some HIV infected people. That’s why many people, these days, also fear testing and coming out in the society”.* - A health worker without HIV, aged 24-years old.

##### Verbal and physicial assualt

Some participants shared their stories of being shamed, being blamed for the transmission of HIV and being beaten by the family members. One community health worker living with HIV shared an experience of being blamed for spreading HIV to the community members.

##### Denial from love and care

Some men living with HIV reported getting divorced from their wives after the disclosure. One woman living with HIV described that her husband was not provided the necessary care in the family and was left to die when he was terminally ill. A few participants shared experiences of women diagnosed with HIV being denied to give childbirth in the hospital and children living with HIV being given nicknames and not allowed to play with other kids in the school.

##### Cultural discrimination*

Stories of cremation rituals not being performed for the people dying of HIV/AIDS were shared by some participants. One woman explained that the dead body of her HIV infected husband remained untouched for several days and cremation ritual was not followed. It was believed that, the dead body of an HIV infected person should not be cremated, because the cremation could spread the disease to other people. She put it this way,

*“(..) the body remained untouched for a couple of days. Later, the villagers decided to dump the body under the soil, but no one was there to touch the body. Eventually, the son from my husband’s first wife and my father tied the body in a bed sheet; and dumped it under the soil near by the house. The villagers even did not put the soil fearing that it transmits that way.”*

“No! no! that’s (the improper cremation) not going to happen to me after I am dead. My body must be cremated properly, following all the rituals.* For this, I am not going to tell anyone, anything about my HIV status, never!” - A man living with HIV, aged 35-years old.

[*According to the Hindu ritual, the dead body should be burned. Burying the body is regarded bad. In past days, the people who died of leprosy or people with some disabilities were buried.]

#### Negative emotions

Several participants agreed having experienced negative emotions related to HIV. For example, a few community members reported perceiving threat of HIV and avoiding PLWH. They imagined that other community members who were vulnerable to HIV might avoid HIV testing uptake because of perceived threat of testing positive and being labelled as a person with HIV and being regarded impure in the community.

Several participants living with HIV who had experienced discriminations perceived themselves contemptuously being a member of an HIV infected group. They were ashamed of talking about their HIV positive status and admitting that they had had extramarital sex. They reported suffering from emotional distress and had been isolating themselves in the community, and one health worker also shared a story of a woman with HIV attempting suicide.

Some of the participants with HIV reported not disclosing the information to others because of the perceived threat of disclosure without consent and discrimination. A few also explained that they did not trust their spouse and family members for keeping HIV information a secret. One political leader diagnosed with HIV (aged 35-years old) put it this way,

*“I haven’t told to anyone about it (HIV status). For instance, I am a politician; people of other parties and within my own party, are looking for this sort of negative things to pull me down. Besides, I am the person who performs all the cultural rituals in the community. My father was the chairperson of the village development committee, one of the most reputed persons in the community. And then, I am infected with this (HIV), how could I talk about it.”*

#### Forced disclosure

Forced disclosure is the process of disclosing one’s HIV status to others by a third party without his/her consent, leading to the spread of HIV information in the community. Several participants reported that the information about their HIV positive status was disclosed to the community members by a health worker, family member or close friend without their consent. They explained that one way of spreading the information about their HIV positive status in the community was via gossiping. Besides, one community health worker living with HIV admitted that he used to tell the community members about PLWH because he had to show that his organization had been doing good for the community. He (31-years old) put it this way;

*“People ask us about HIV infected people. (…). We should say the truth because we need to make people recognize our (organization’s) work.”*

*“If one person finds about it (HIV status), then it gets spread everywhere and everyone will know about it. After everyone knows about it, there's no such thing to hide or keep a secret.”* - One woman living with HIV, 28-years old.

Several PLWH explained engaging in strategies to make sure that the community members would not find out about their HIV positive status. For instance, some PLWH reported telling lies. A few reported using jargon such as ‘bad blood’ or ‘jaundice’ or ‘a new disease’ or ‘little disease’ instead of HIV. The ART counsellor explained that some of his patients provided fake information about personal data and insisted not to have an appointment in public and not to reveal the information to others. Some participants imagined that, because of the fear that the HIV information gets spread, some people may avoid seeking care in the hospital and may instead seek help from traditional healers. One widowed women (aged 35-years-old) stated,

*“My husband fell sick after he returned from India. He had high fever and cough and was eating nothing. I asked him to go to the hospital for several times, but he was never ready for it at all. We went seeking help from many traditional healers and none of them worked out. (…). He might have already known that he had AIDS.”*

### Public health system to control HIV

A public health system is defined as the spectrum of public health initiatives that are delivered to control the HIV epidemic and to insure health and general welbeing of PLWH. These public health intiatives include delivery of general and HIV-specific health care, provision of incentives to increase access to health services and actively involving PLWH in the community. These initiatives are associated with increasing knowledge, changing perception and establishing a good relationship with health workers, which would eventually empower PLWH. This system is primarily based on the dynamic and positive relationship between empowerment and self disclosure. For example, this system views empowerment as a mechanism and also as an outcome of self-disclosure. Increases in self-disclosure rates is a means to prevent new transmissions in the community in this system. Each component of this system is described below:

#### Public health initiatives

The majority of the participants explained that free access to treatment, testing and prevention messages and other general health services, such as in-patient services, maternity services and family planning services, had been increasing over the years. All the health workers reported that various incentives, such as transportation allowances, free meals and lodging facilities, had also been provided to increase people’s access to prevention, treatment and social support services.

A health worker explained that, in some places, the community organizing initiatives for PLWH had been very effective. He gave an example of community micro-financing groups, which had been actively providing loans for income generating activities to PLWH. Several participants agreed that the PLWH had been encouraged to involve in community-level activities, such as community development, community organization, HIV-related activities, health service delivery and income generation activites.

*“We have made three committees of PLWH in three different places. We provide $400 per year to each committee. Members from each committee can take a loan and use the fund to run their own businesses.”* – A health worker living with HIV, aged 28-years old.

#### Mechanisms to empowerment

The majority of participants living with HIV agreed that access to public health services had led to changes in knowledge and perception and established a positive relationship with health workers. They explained that, because of access to treatment and health services, they were aware of benefits associated with HIV disclosure. They believed that increased access to treatment and health services were the reasons for living longer and healtheir life, giving birth to children and enjoying a family life. Some of them also believed that access to treatment led to family care and support and helped in networking with other PLWH. A few participants including one health worker reported that community and political involvement had helped to strengthen their knowledge on policy issues, upgraded their skills to navigate in political spaces, and enhanced their strategies for networking with other people.

A few participants living with HIV reported being engaged in a job or business or having a regular income, being involved in community organizations or community development intitiatives, including school management, construction of roads and provision of health care. They revealed being able to introduce themselves as an HIV infected person, raise voice for inclusion of PLWH in community development initiatives, help other people to open up about their HIV status and work to maintain the solidarity among the groups of PLWH. A community health worker reported that other people regard him as a role model for publicly disclosing and actively involving in community development activities. Several other participants also shared similar examples of other PLWH in their community being actively involved in planning and implementation of community-level activities and getting jobs and having an income. A few imagined that they could also involve in an organization, have a good income, fight against all forms of discriminations and be respected in the community.

*“While visiting the community, we carry the bags having an official logo of our organization. People who notice that logo would definitely know about us that we are people with HIV and work for an HIV-related organization. One or two people had also asked me about me and why I come to their village. Some people already know me as an HIV infected person.” –* A community health worker living with HIV, aged 31-years old.

#### Self disclosure

Self-disclosure of HIV is a process of communication by which one person reveals information about himself or herself to another. However, several participants revealed being unable to make decisions whether, how and whom to share this information with. A few believed that self-disclosure would be easier with health workers, educated family members, members of a younger generation, and other HIV positive people.

People diagnosed with HIV take some time to gather sufficient knowledge and make decisions about how and whom to share the information about an HIV positive status. They often start sharing information with a limited selection of individuals (e.g. health workers, family members and close friends) and slowly, this process can lead to public disclosure. Some PLWH mentioned disclosing to a health worker first. Some women reported telling their mother and some their eldest son. Some participants reported seeking help from health workers to inform their spouse and family members about their HIV positive status.

### Interaction between the community self-coping with HIV and public health system to control HIV

Although the ultimate aim of a community self-coping system and a public health system is to control the transmission of HIV in the community, the mechanism of action differs between both. A community that is self coping with HIV seeks to control HIV via an increased level of forced disclosure leading to an exclusion of PLWH from the community. While a public health system seeks to do it via the promise of empowering PLWH hereby stimulating self-disclosure to gain access to the system.

The society has to deal with both driving forces in terms of behaviours and practices. For instance, in a community self-coping system, misconceptions and perceived threat of HIV lead to avoidance of HIV prevention services (e.g. HIV test uptake) and non-disclosure. A public health system, on the contrary, would encourage people to access public health services and to disclose an HIV positive status via motivational health-related messages and incentives. The dichotomy of disclosure or non-disclosure behaviour is influenced by two driving forces, that is perceived threats in a community self-coping system and perceived benefits in a public health system. In case, the force from a public health system is stronger, people’s disclosure status is subject to move from undisclosed to disclosed status.

These two driving forces have an influence on the outcomes of the disclosure. For example, in a community self-coping system, forced disclosure is often linked with negative outcome experiences (e.g. exclusion); whereas in a public health system, self-disclosure is often linked with positive outcome experiences (e.g. access to treatment). Depending on which force is stronger, PLWH may have both positive and negative experiences after the disclosure. For example, some PLWH who are disclosed without consent may later on be encouraged by a health worker or a family member to gain access to a public health system. On the other hand, among the people who have disclosed themselves, some may be accepted in the community, while some may still be subject to exclusions. Those who are accepted in the community may work as advisors and may motivate others to self-disclose and to gain access to public health system.

A public health system may have an impact on the stigma reduction process. For instance, increased access to HIV-related messages may directly lead to changes in knowledge and perception of the community people. In this case, people diagnosed with HIV may find it easier to self-disclose and gain access to public health system. Increased self-disclosure rates and increased visibility of PLWH in the community, may further lead to changes in knowledge and perception of the community members and may challenge the existing HIV-related taboos and misconceptions.

## Discussion

Our theoretical model illustrates that, in a community self-coping system, the stigmatization of HIV leads to forced disclosure because of perceived threat and social curiosity among the community members without HIV. This is the central mechanism for enforcing social norms to distance PLWH from the community members. The public health system emphasizes collective empowerment as the central mechanism to promote self-disclosure among PLWH. A public health system has an impact on community self-coping system by reducing social stigma via increasing knowledge and changing perception of the community people. This may encourage people diagnosed with HIV to disclose themselves about their HIV status and gain access to the system.

Our theoretical model illustrates that, in a community self-coping system, community members may see stigmatizing PLWH as normal behavior in order to control the transmission. However, this system may exacerbate the perceived threat of community attitudes, increase non-disclosure of HIV and consequently, increase the rate of HIV transmission among at-risk populations via engaging in risk practices. At an individual level, forced disclosure and discrimination ultimately provide PLWH with limited access to resources, such as social, cultural and medical. This has a great impact on their lives and their chances to survive [25]. Thus, without reducing stigma and potential harms to PLWH, encouraging disclosure may have more harmful effects at the social and individual level.

A public health system often ignores community-based indigenous and traditional systems. Nevertheless, health programs that do not recognize and work with community beliefs and practices may fail to reach their goals. For example, one of the reasons for low uptake of HIV testing and ART services and high discontinuation of ART was due to beliefs and practices-related to traditional health care and perceived dissatisfaction with public health services among PLWH in Kenya (2011) and South Africa (2011) [26, 27]. WHO estimates that, in some countries in Asia and Africa, more than 80% of the population believe and engage in community-based traditional health practices [28]. In practice, for a health care provider, it is important to have a clear picture of traditional community-based traditional beliefs and practices so that s/he can provide appropriate advice on delayed HIV testing or ART dropout [29]. In line with Flint, our framework specifically states that, if we are to achieve universal access to treatment and testing, there needs to be explicit recognition of, and further strategies to counter, the context surrounding the practice of traditional medicine among vulnerable population groups [30].

Several interventions have been conducted to integrate a traditional community system in a broader public health system and a great majority of such approaches have focused training and engaging traditional healers’ as a counsellor or educator in health care [30–33]. However, these approaches have been more effective in some contexts and less in the other. For example, working in Nepal, Poudel and colleagues have reported that, after providing training to traditional healers, they were able to provide culturally acceptable health education to the community people, and played a role in reducing the HIV/AIDS-related stigma via visiting the PLWH in their homes [31].

However, in Zambia, a similar work to integrate traditional healers as a counsellor or educator in HIV prevention and care revealed that the intervention was ineffective to produce the desired outcomes [32]. Mosabela et al. (2016) have stated that some of the traditional healers themselves found it difficult to visit and use HIV clinics due to fear of labelling, stigma and discrimination [34]. Moreover, most patients diagnosed with HIV took seeking care from traditional healers as alternatives to visiting HIV clinics, and traditional healers faced perceived difficulties to channel PLWH into clinics through referrals. In the current study, we also observed that, the community educators, on one hand, were empowering PLWH to disclose their HIV status, and on the other hand, they were also involved in spreading the HIV information without consent of some PLWH, which was associated with negative experiences. Our theoretical model may be used to understand how the community-based values and systems would influence these community-based approaches, and how these approaches would impact the lives and communities of PLWH.

Most of health programs in developing countries are funded by external donor agencies and are fixed-term [35]. They are based on collaborations with local non-governmental organizations or local communities and are meant to be handed over to them at the end. Today, in the field of HIV prevention, most of these collaborations may not be sustainable because of a regression in funding and the challenge to minimize the funding gap to continue providing HIV treatment and services [35, 36]. One fundamental assumption based on our theoretical model could be that, a reduction in number of public health initiatives may exacerbate existing community misconceptions and negative perceptions, which would lead to lower rates of health service access. Unless each aspect of traditional community systems is carefully looked upon, interactive collaboration between traditional community systems and public health, and handing over the public health interventions to local governments and communities may be less effective.

Unlike other models of HIV disclosure, the current model provides new insights in how to approach problematic disclosure processes and potentially develop a useful response in trying to control HIV [2, 3, 7, 9]. With our work, we would like to emphasize that, in terms of designing and developing interventions, community self-coping and public health systems are not completely incompatible but may even be perceived complimentary, and the mechanisms of disclosure in both systems would be important to identify the best strategies to design, plan, implement and evaluate an HIV prevention project. As our theoretical model is comprehensive, it is not necessary that the interventions to reduce stigma and increase disclosure are based on targeting all the aspects of the model. For example, Bassett has noted that the provision of financial incentives is associated with reduced stigma and an increased uptake of HIV testing, care, and prevention services [37]. Besides, how each intervention would independently lead to reduced stigma levels and consequently, high HIV testing uptake and high disclosure rates may be subject to further exploration [38].

One of the strengths of our study is that we opted for a grounded theory design using an inductive and iterative approach to select and interview the participants and to develop the theoretical model. One example of following an iterative process is that, we started with one-to-one interviews and found PLWH being uncomfortable while talking about their personal experience. We then invited two or three PLWH to participate in a group interview. Interestingly, we found that group interviews compared with one-to-one interview were more effective for an insightful discussion on disclosure experiences. It might be that, in a group situation, the information that is revealing would be common to all the members in terms of their experiences, which makes it a matter of discussion rather than the disclosure of individual stories. Moreover, we composed the group in a way that there was similarity among all the members in terms of their social background that may have lead to an insightful discussion.

This study has two major limitations. First, only the PLWH who were enrolled for treatment were recruited. It would have been better if we could have included other people diagnosed with HIV who had not disclosed their status, not even to a health worker. These people may experience things differently than the people who have already disclosed to a health worker. However, it is not ethically permitted to identify and talk to the people who have not disclosed their HIV status with their consent. Second, because we only included the health workers living with HIV or working for PLWH, it is possible that these health workers were likely to report positive attitudes about HIV. Given that the culture of health care system also plays an important role in a disclosure process [25], disclosure issues and stigma experiences in health care settings were underappreciated in this study. Although the field work for this study was based among at-risk population in the Achham district of Nepal, it would be interesting to test the theory in similar settings and populations in other regions to evaluate whether the theoretical model is applicable.

## Conclusions

Our theoretical model illustrates how two dominant systems to control HIV, namely a community self-coping and a public health system, independently or jointly, shape contexts, mechanisms and outcomes for HIV disclosure. We believe, this model will be of theoretical as well as of practical relevance in advancing the field.

First, on a theoretical level, our study adds to research on developing collaborative approaches with traditional health practices and how these collaborations would impact the lives and communities. Second, the model emphasizes that a public health system is crucial for stigma reduction. Researchers may be interested to study how public health interventions would lead to reduced stigma levels and increased HIV test uptake and disclosure rates [38].

On a practical level, our theoretical model provides a robust account of PLWH’s experiences with a community self-coping and a public health system and emphasizes to carefully look upon all aspects of both systems while developing and designing any HIV interventions [28]. With our work, we want to draw the attention of public health practitioners to understand and address the context surrounding the practice of traditional medicine among vulnerable populations while implementing public health interventions to achieve universal access to treatment and testing.

## Abbreviations

ART: Anti-retroviral treatment
HIV/AIDS: Human immunodeficiency virus and acquired immune deficiency syndrome
PLWH: People living with HIV
UNAIDS: Joint United Nations Program on HIV/AIDS
WHO: World Health Organization
